# Impact on 6-month outcomes of hospital trajectory in critically ill older patients: analysis of the ICE-CUB2 clinical trial

**DOI:** 10.1186/s13613-022-01042-4

**Published:** 2022-07-11

**Authors:** Sara Thietart, Ariane Boumendil, Dominique Pateron, Bertrand Guidet, Hélène Vallet, the ICE-CUB2 Study Network, Bertrand Guidet, Dominique Pateron, Erwan Debuc, Youri Yordanov, Ariane Boumendil, Caroline Thomas, Didier Dreyfuss, Jean-Damien Ricard, Patrick Brun, Christophe Leroy, Yves Cohen, Frédéric Adnet, Maguy Woimant, Jean-Paul Mira, Benoît Doumenc, Khalil Tku, Thomas Similowski, Bruno Riou, Pierre Hausfater, Samuel Delerme, Jean-Pierre Quenot, Didier Honnart, Jean-François Timsit, Pierrick Guérin, Françoise Carpentier, Maxime Maignan, Foued Makhlou, Jean-François Poussel, Yohann Picard, François Braun, Pauline Trognon, François Fourrier, Patrick Goldstein, Marie Girot, Pierre Gosselin, Francesco Santoli, Pierre Charestan, Claire Poly, Hervé Mentec, Catherine Le Gall, Karima Sahraoui, Christophe Baillard, Nicolas Javaud, Benoît Misset, Maité Garrouste-Orgeas, Olivier Ganansia, François-Xavier Rooryck, Jean Luc Aim, Abudlrazak El Rifai, Jean Reignier, Laurent Martin-Lefevre, Philippe Fradin, Claire Mauriat, Emelyne Cwicklinski, Michel Slama, Hervé Dupont, Christine Ammirati, Justine Gallou, Muriel Fartoukh, Michel Djibre, Patrik Ray, Edwin Rouff, Bertrand Souweine, Ali Ait Hssain, Jeannot Schmidt, Daniel Pic, Farès Moustafa, Alain Mercat, Nicolas Lerolle, Pierre-Marie Roy, Frédéric Baud, Patrick Plaisance, Sophie Montagnon, Bertrand Galichon, Michel Wolff, Bruno Mourvillier, Enrique Casalino, Christophe Choquet, Julien Bernard, Gaëlle Juillien, Jean-Yves Fagon, Emmanuel Guerot, Philippe Juvin, Anabela Patzak, Bruno Verdière, Vincent Ioos, Marie-Clément Kouka, Audrey Berthoumieu, Christian Richard, Raphael Maurice, Sophie Sarnel, Stéphane Diez, Antoine Vieillard Baron, Sébastien Beaune, Julie Grenet, Sylvie Azerad, Guillaume Leblanc, Tabassome Simon

**Affiliations:** 1 Department of Intensive Care, APHP, Hôpital Saint-Antoine, Sorbonne Université, 184, rue du Faubourg Saint-Antoine, 75012 Paris, France; 2 EBMT LWP, Paris Office, Hôpital Saint-Antoine, Paris, France; 3 Department of Emergency, APHP, Hôpital Saint-Antoine, Sorbonne Université, Paris, France; 4 INSERM, Institut Pierre Louis d’Epidémiologie et de Santé Publique, APHP, Hôpital Saint-Antoine, Paris, France; 5 Department of Geriatrics, APHP, Hôpital Saint-Antoine, Sorbonne Université, Paris, France; 6 INSERM U1135, Centre d’Immunologie et des Maladies Infectieuses (CIMI-Paris), Paris, France

**Keywords:** Geriatrics, Outcome, Functional status, Intensive care, Critical care

## Abstract

**Background:**

Little is known about the impact of hospital trajectory on survival and functional decline of older critically ill patients. We evaluate 6-month outcomes after admission to: intensive care units (ICU), intermediate care units (IMCU) or acute medical wards (AMW).

**Methods:**

Data from the randomised prospective multicentre clinical trial ICE-CUB2 was secondarily analysed. Inclusion criteria were: presenting at emergency departments in critical condition; age ≥ 75 years; activity of daily living (ADL) ≥ 4; preserved nutritional status; and no active cancer. A Cox model was fitted to compare survival according to admission destination adjusting for patient characteristics. Sensitivity analysis using multiple imputation for missing data and propensity score matching were performed.

**Results:**

Among 3036 patients, 1675 (55%) were women; median age was 85 [81–99] years; simplified acute physiology score (SAPS-3) 62 [55–69]; 1448 (47%) were hospitalised in an ICU, 504 in IMCU (17%), and 1084 (36%) in AMW. Six-month mortality was 629 (44%), 155 (31%) and 489 (45%) after admission in an ICU, IMCU and AMW (*p* < 0.001), respectively. In multivariate analysis, AMW admission was associated with worse 6-month survival (HR 1.31, 95% CI 1.04–1.63) in comparison with IMCU admission, after adjusting for age, gender, comorbidities, ADL, SAPS-3 and diagnosis. Survival was not significantly different between patients admitted in an ICU and an IMCU (HR 1.17, 95% CI 0.95–1.46). Sensitivity analysis using multiple imputation for missing data and propensity score matching found similar results. Hospital destination was not significantly associated with the composite criterion loss of 1-point ADL or mortality. Physical and mental components of the 12-Item Short-Form Health Survey were significantly lower in the acute medical ward group (34.3 [27.5–41.7], *p* = 0.037 and 44.3 [38.6–48.6], *p* = 0.028, respectively) than in the ICU group (34.7 [28.4–45.3] and 45.5 [40.0–50.0], respectively) and IMCU group (35.7 [29.7–43.8] and 44.5 [39.7–48.4], respectively).

**Conclusions:**

Admission in an AMW was associated with worse 6-month survival in older critically ill patients in comparison with IMCU admission, with no difference of survival between ICU and IMCU admission. There were no clinically relevant differences in quality of life in each group. These results should be confirmed in specific studies and raise the question of dedicated geriatric IMCUs.

**Supplementary Information:**

The online version contains supplementary material available at 10.1186/s13613-022-01042-4.

## Background

The proportions of older patients hospitalised in intensive care units (ICU) are increasing due to ageing populations [1–3]. Older patients present specific conditions, such as frailty, polypharmacy and multimorbidity, making it more difficult for them to overcome acute medical stress and to survive and recover their previous functional autonomy [4, 5]. For all these reasons, mortality is increased among older patients hospitalized in an ICU, comparatively with younger populations, with a 6-month mortality ranging from 21 to 58% [1, 6–8]. Most studies evaluating short- and long-term mortality are heterogeneous in design and results, and factors associated with mortality vary widely across studies [9]. Outcomes of older patients hospitalised in ICUs are influenced by premorbid conditions, notably frailty and comorbidities, as well as in-ICU events, such as duration of mechanical ventilation and decisions to withdraw life-sustaining therapies [4, 5, 9–13]. In addition, among survivors, some may suffer from disabilities, cognitive impairment and decreased quality of life [14, 15].

Currently, the benefits on mortality and qualitative outcomes (functional status and quality of life) of an ICU hospitalisation for a critically ill older patient are unclear. The question of which structure is best adapted to the needs of these patients has yet to be answered. We previously demonstrated in the randomised clinical trial ICE-CUB2 that promoting systematic ICU admission of critically ill older patients had no effect on mortality in comparison with standard practice, but analysis of the impact of hospitalisation in intermediate care units was not evaluated [16]. Intermediate care units (IMCUs) are used to manage patients needing more care than a general ward, but requiring lighter monitoring than ICUs, which, theoretically, have more severely ill patients [17]. Conflicting results are found in studies evaluating the effect of IMCU hospitalization on mortality [17–21], and very few studies were performed specifically on older populations [22, 23].

We, therefore, aim to evaluate whether the structure in which the older critically ill patient is hospitalised influences longer term outcomes.

## Methods

### Study design

All patients were included in a previously published cluster-randomised prospective multicentre clinical trial conducted in 24 academic and non-academic hospitals in France from January 2012 to November 2015 (NCT01508819) [16]. The trial conformed to the ethical guidelines of the Declaration of Helsinki and was approved by the Institutional Review Boards (CPP Ile-de-France 9).

### Patient selection

Patients were included if they were aged 75 years or older, with a preserved functional status (defined by a Katz Index of Independence in activity of daily living (ADL) of at least 4) [24], a preserved nutritional status (defined as the absence of cachexia, subjectively evaluated at bedside by a physician), with no active cancer, who arrived in an emergency department with a pre-established critical condition, as listed in ICE-CUB2 and were then hospitalised either in an ICU, an IMCU, or a standard acute medical ward (AMW) [16].

### Data collection

Functional ability was evaluated using an ADL score, and quality of life using the physical and mental components of the 12-Item Short-Form Health Survey (physical SF-12 and mental SF-12) scale [25]. Data on age, sex, comorbidities, living situation, home support, Simplified Acute Physiology Score 3 (SAPS-3) [26], ADL score at inclusion, initial clinical diagnosis, length of stay, type of ward (ICU, IMCU or AMW), hospital discharge location and in-hospital mortality were assessed. Mortality, ADL, physical SF-12 and mental SF-12 were assessed at 6 months by phone-calls.

### Definitions

An ICU was defined as a ward providing intensive and specialised medical and nursing care, with an enhanced capacity for monitoring, and that can provide organ support for multiple life-threatening organ insufficiencies [27]. An IMCU was defined as a ward providing close patient monitoring to manage patients with unique life-threatening organ insufficiencies, with a lower nurse-to-patient ratio than in an ICU [17]. Both general and specialized high dependency units could be classified as IMCUs. An AMW was defined as a ward with a low nurse-to-patient ratio, with a daily medical evaluation but without continuous monitoring. These wards admit patients with organ insufficiencies but with life-sustaining treatment (LST) limitations.

### End-points

Primary end-point was overall survival 6 months after admission in any of the three structures: ICU, IMCU, or an AMW. Secondary outcomes were (1) factors associated with 6-month survival, (2) factors associated with the occurrence of the composite criterion death or loss of functional ability 6 months after admission, and (3) quality of life 6 months after admission using physical SF-12 and mental SF-12.

### Statistical analysis

Baseline characteristics of patients were analysed as frequencies and percentages for categorical variables and as medians and interquartile ranges for continuous variables. Comparisons between admission location were evaluated using the Kruskal–Wallis test (ANOVA) for continuous variables and the *χ*^2^ or Fisher exact test for categorical variables as appropriate. Binary outcomes (death or change in ADL) were analysed using logistic regression models. To compare survival according to admission location adjusting for patient characteristics, a Cox model was fitted including the following variables: age, gender, invalidating illness, baseline ADL, SAPS3, and admission diagnosis. Furthermore, to investigate whether factors influencing outcomes were different according to admission location, separate models were fitted for each admission location and each outcome (death or change in ADL, 6-month survival). No assumption or imputation of missing data was performed for the primary analysis. A secondary analysis was performed using multiple imputation for missing values to test robustness of the model and the findings. Sensitivity analysis using propensity score were performed to check reliability of results. Two 1:1 propensity score matching (PSM) analysis were used to minimize covariate differences between ICU and IMCU, and AMW and IMCU, respectively. Propensity score was estimated with a binary logistic regression including the following covariates: age, gender, invalidating illness, autonomy, severity and admission diagnosis, living situation, experience of the emergency department physician, experience of the intensive care physician; patients were then matched using the nearest neighbour matching based on the propensity score. All analyses were performed at a two-sided alpha level of 5%. A *p* value of less than 0.05 was considered to indicate statistical significance. All analyses were performed (AB) with R statistical software, version 3.2.2 (R Foundation for Statistical Computing).

## Results

### Study population

Among the 3036 patients presenting in an emergency department for a critical condition [16], 1448 (47%) were hospitalised in an ICU, 504 (17%) in an IMCU and 1084 (36%) in an AMW. Baseline characteristics according to ward destination are described in Table 1. Median age was 85 [81–89] years, 1675 (55%) were female, and median SAPS-3 was 62 [55–69]. The three most frequent clinical diagnosis were respiratory failure (*n* = 949, 31%), shock (*n *= 558, 18%) and cardiac disorders (*n* = 408, 13%). Main comorbidities were ischemic heart disease or hypertension (*n* = 854, 42%), respiratory disorders (*n* = 632, 31%), and congestive heart failure (*n* = 270, 13%). Most patients were living at home (*n* = 2600, 86%) and the median ADL score at admission was 6 [5.5–6].

**Table 1:** Patient characteristics

	Intensive care unit	Intermediate care unit	Acute medical ward	*p* value
*N*	1448	504	1084	
Age (years)	84 [80–88]	85 [81–89]	87 [83–92]	< 0.001
Female sex	764 (53)	259 (51)	652 (60)	< 0.001
Comorbidities^a^				< 0.001
Ischemic heart disease or hypertension	375 (40)	190 (51)	289 (39)	
Respiratory disorder	286 (31)	97 (26)	249 (34)	
Congestive heart failure	123 (13)	60 (16)	87 (12)	
Neurological disorders	105 (11)	34 (9)	83 (11)	
Cognitive impairment	94 (10)	33 (9)	126 (17)	
Cirrhosis	23 (3)	4 (1)	5 (1)	
ADL at inclusion^b^	6 [5.5–6]	6 [5.5–6]	6 [5–6]	< 0.001
Living situation at inclusion^c^				< 0.001
Home without assistance	1049 (72)	354 (70)	635 (58)	
Home with assistance	243 (17)	83 (17)	236 (22)	
Long-term care facility	78 (5)	21 (4)	95 (9)	
Nursing home	70 (5)	37 (7)	99 (9)	
Other	8 (1)	9 (2)	19 (2)	
SAPS-3^d^	64 [57–71]	57 [52–63]	59 [54–67]	< 0.001
Admission diagnosis^e^				< 0.001
Respiratory failure	477 (33)	124 (25)	378 (35)	
Shock	371 (26)	37 (7)	150 (14)	
Coma	142 (10)	26 (5)	151 (14)	
Cardiac disorders	136 (9)	151 (30)	121 (11)	
Acute kidney failure	91 (6)	19 (4)	37 (3)	
Gastrointestinal tract disorder	77 (5)	31 (6)	66 (6)	
Surgery	30 (2)	2 (0)	30 (3)	
Multiple trauma without surgery	10 (1)	4 (1)	5 (1)	
Other causes	112 (8)	110 (22)	145 (13)	

Continuous variables are expressed as median [1st–3rd quartiles], and categorical variables as sample size/missing data (percentages). Neurological disorders are defined as premorbid stroke or Parkinson’s disease. Missing values: ^a^513 for ICU, 128 for IMCU, 343 for acute medical ward; ^b^206 for ICU, 66 for IMCU, 235 for acute medical ward; ^c^0 for ICU, IMCU and acute medical ward; ^d^66 for ICU, 53 for IMCU, 65 for acute medical ward; ^e^2 for ICU, 0 for IMCU, 1 for acute medical ward

### Survival according to admission location

**Fig. 1:**
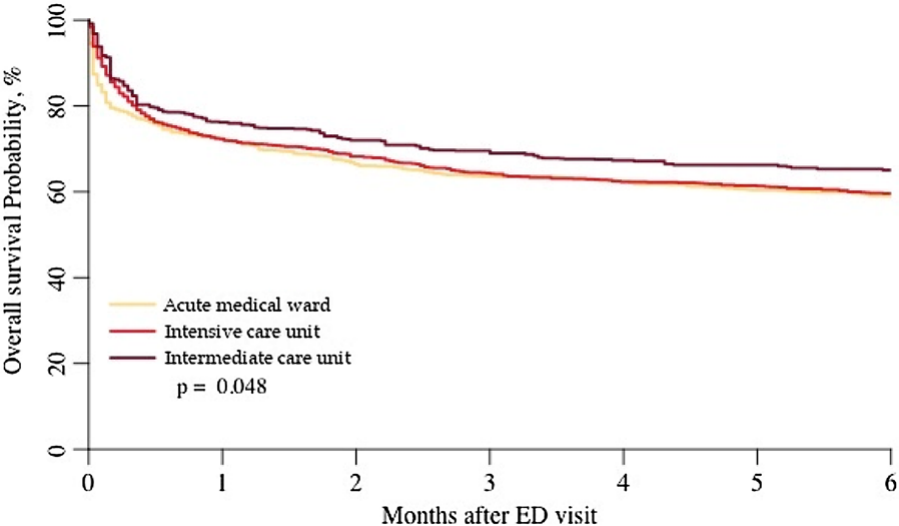
Adjusted 6-month overall survival according to ward destination in older critically ill patients

In-hospital mortality was 409 (28%) for patients hospitalised in an ICU, 77 (15%) in an IMCU and 291 (27%) in an AMW. 6-month mortality was 629 (44%) when admitted in an ICU, 155 (31%) in an IMCU and 489 (45%) in an AMW (*p* < 0.001). In multivariate analysis, admission in an AMW (HR 1.31, 95% CI 1.04 to 1.63) was associated with worse overall survival than admission to an IMCU. Other factors associated with worse survival were age (HR 1.04 for each 1-year increase, 95% CI 1.03 to 1.05), male gender (HR 1.19, 95% CI 1.04 to 1.36), ADL (HR 1.15 for each one-point decrease, 95% CI 1.06 to 1.25), SAPS-3 (HR 1.05 for each one-point increase, 95% CI 1.04 to 1.05) and diagnosis of coma (HR 1.56, 95% CI 1.21 to 2.02) (Table 2). Survival probabilities according to destination are shown in Fig. 1.

**Table 2:** Factors associated with 6-month overall survival—multivariate analysis

Variables	Hazard ratio (95% CI)^a^	*p* value
Hospital destination (vs IMCU)		
AMW	1.31 (1.04–1.63)	0.019
ICU	1.17 (0.95–1.46)	0.147
Age (per one-point increase)	1.04 (1.03–1.05)	< 0.001
Male sex (vs female)	1.19 (1.04–1.36)	0.012
Presence of comorbidities	1.04 (0.90–1.20)	0.562
SAPS-3 (per one-point increase)	1.05 (1.04–1.05)	< 0.001
ADL (per one-point decrease)	1.15 (1.06–1.25)	< 0.001
Admission diagnosis (ref: cardiac disorder)		
Surgery	0.97 (0.59–1.61)	0.909
Coma	1.56 (1.21–2.02)	< 0.001
Respiratory failure	0.92 (0.74–1.15)	0.465
Gastrointestinal tract disorder	0.67 (0.46–0.97)	0.034
Shock	0.80 (0.62–1.01)	0.065
Multiple trauma with no surgery	1.08 (0.44–2.65)	0.870
Acute kidney injury	0.97 (0.69–1.36)	0.865
Other	0.79 (0.59–1.05)	0.102

Multivariate analysis using Cox model with the following variables: SAPS-3, gender, age, admission diagnosis, invalidating illness, baseline ADL, initial diagnosis

95% CI: 95% confidence interval, ADL: Activity of daily living, ICU: intensive care unit, IMCU: intermediate care unit, HR: hazard ratio, ref: reference, SAPS-3: Simplified Acute Physiology Score 3

^a^HR gives the increase of the risk of death per each unit increase for continuous variables and for one specific category vs a reference category for categorical variables (HR > 1: the variable is associated with an increased risk of death or decreased survival)

Furthermore, age and ADL were significantly associated with overall survival for patients hospitalised in ICUs or IMCUs but not for patients hospitalised in AMWs (Additional file 1: Table S1).

### Mortality or loss of 1-point ADL

The median change in ADL between admission and 6 months after admission was − 0.5 for all 3 destinations [IQR − 1.5–0 for ICU and IMCU; IQR − 2–0 for AMW], *p* = 0.374. In multivariate analysis, hospital destination was not associated with the occurrence of the composite criterion consisting of mortality or loss of 1-point ADL, as shown in Table 3. Factors associated with mortality or loss of 1-point ADL were comparable in each destination, except for gender and diagnosis of coma, respiratory failure and shock (Additional file 1: Table S2).

**Table 3:** Multivariate analysis of factors associated with mortality or loss of one-point ADL at 6 months

Variables	Odds ratio (95% CI)^a^	*p* value
Hospital destination (ref IMCU)		
AMW	1.05 (0.99–1.11)	0.096
ICU	1.05 (0.99–1.11)	0.105
Age (per one-point increase)	1.01 (1.01–1.02)	< 0.001
Male sex (vs female)	0.98 (0.95–1.02)	0.405
Presence of comorbidities	1.04 (1.00–1.09)	0.041
SAPS-3 (per one-point increase)	1.01 (1.01–1.01)	< 0.001
ADL (per one-point decrease)	1.04 (1.01–1.07)	0.007
Admission diagnosis (ref: cardiac disorder)		
Surgery	0.98 (0.85–1.13)	0.754
Coma	1.1 (1.01–1.19)	0.025
Respiratory failure	0.99 (0.93–1.05)	0.721
Gastrointestinal tract disorder	0.94 (0.85–1.04)	0.206
Shock	0.91 (0.85–0.98)	0.011
Multiple trauma with no surgery	0.90 (0.71–1.14)	0.373
Acute kidney injury	0.96 (0.87–1.05)	0.369
Other	0.91 (0.84–0.98)	0.009

Multivariate analysis using logistic regression model

95% CI: 95% confidence interval, ADL: Activity of daily living, ICU: intensive care unit, IMCU: intermediate care unit, OR: odds ratio, ref: reference, SAPS-3: Simplified Acute Physiology Score 3

^a^OR gives the factors associated with overall 6-month mortality or loss of 1-point ADL per each unit increase for continuous variables and for one specific category vs a reference category for categorical variables

### Quality of life 6 months after admission

Among 1654 survivors with available follow-up data, living situations at 6 months were: at home without assistance for 740 patients (45%); at home with assistance for 542 patients (33%); and nursing homes for 268 patients (16%). Living situations 6 months after admission did not differ according to hospital destination. Median 6-month physical SF-12 was higher among patients admitted to an IMCU (35.7 [29.7–43.8]), than an ICU (34.7 [28.4–45.3]) or an AMW (34.4 [27.5–41.7]; *p* = 0.037). 6-Month mental SF-12 was highest when admitted in an ICU, and lowest when hospitalised in an AMW (45.5 [40.0–50.0] in ICU, 44.5 [39.7–48.4] in IMCU and 44.3 [38.6–48.6] in AMW; *p* = 0.028).

### Sensitivity analysis

Using multiple imputation for missing data, admission to an AMW was still associated with worse overall 6-month survival (HR 1.35, 95% CI 1.11 to 1.63), as shown in Additional file 1: Table S3. After matching on the propensity score, AMW admission was associated with a worse 6-month overall survival than admission to an IMCU (HR 1.76, 95% CI 1.23 to 2.53). Admission in an ICU was also associated with a worse survival (HR 1.5, 95% CI 1.07 to 2.10) in comparison with being admitted in an IMCU, as shown in Additional file 1: Table S4. Admission in an AMW or an ICU were associated with higher risks of death or loss of one-point ADL at 6 months, in comparison with IMCU admission, after propensity score matching: HR 1.12 (95% CI 1.03 to 1.22) and HR 1.15 (95% CI 1.06 to 1.26), respectively (Additional file 1: Table S5).

## Discussion

Although patient characteristics and severity differed according to ward destination, patients hospitalised in an AMW had a significantly worse 6-month survival than those admitted to an IMCU, after adjusting for baseline characteristics, severity and diagnosis, and propensity score matching. No significant differences in survival were found between patients admitted to an ICU and an IMCU. Hospital destination was not associated with the composite outcome of 6-month mortality or loss of functional ability. Mental and physical SF-12 was lowest in patients hospitalised in an AMW, although the difference was not clinically relevant.

Six-month mortality after IMCU admission was 31%, which is similar to the findings of other studies [22, 23, 28, 29]. Survival was significantly worse when hospitalised in an AMW in comparison with IMCU admission, with no difference of survival between patients hospitalised in ICUs and IMCUs. The three groups differ in comorbidities, functional ability, diagnosis and severity. These differences of comorbidities and severity could be explained by the fact that patients eligible for IMCUs have a unique life-threatening organ insufficiency [17], whereas patients with multiple organ failure were admitted in an ICU, or an AMW when ICU admission was refused because of life-sustaining treatment limitation. However, when adjusting on age, severity, comorbidities and diagnosis, we found that admission in an IMCU was still associated with a better survival than in an AMW, with no significant difference with ICU admission. These results were confirmed after propensity score matching, where admission to an IMCU was associated with better survival in comparison with AMW and ICU admission. In the context of aging of population, these results suggest that there could be a benefit of hospitalising older patients in IMCUs, notably in geriatric IMCUs [30].

The SARS-CoV-2 pandemic has shown the importance of expanding critical care capacity, notably by creating IMCUs [31–34]. With the aging of population, it is expected that by 2070, 20% of the French population will be aged 75 years and over [30]. It is thus a possibility that tension in critical care capacity could occur again, even without a pandemic. An interactive session of the 2019 European Society of Intensive Care Medicine congress suggested that progress on pre-ICU triage and creation of dedicated intermediate care units for critically ill very old patients could be solutions to explore [35]. These IMCUs can offer monitoring for less severe critically ill patients, or facilitate ICU discharge as a “step-down unit”, thereby freeing up ICU beds [36]. Our study found better survival when patients with critical conditions were hospitalised in an IMCU than in an AMW, with no difference when admitted in an ICU. Thus, our results could suggest that there is no loss of opportunity for the older patient between being admitted in an ICU or an IMCU.

SAPS-3 was significantly associated with poorer outcomes independently of hospital destination. No score predictive of mortality among critically ill patients was created specifically for older patients, mean age at inclusion in the SAPS-3 study being only 63 years [26]. SAPS-3 is a score predicting hospital mortality, but contains no variables specific to older patients, such as functional status. We found that baseline ADL was associated with poorer outcomes only in patients hospitalised in ICUs and IMCUs, but not in AMWs. This could suggest that one should admit in an ICU or IMCU mainly patients with preserved functional ability. Other factors specific to older patients are known to influence ICU outcomes, such as the Clinical Frailty Scale, its components being items reflecting functional ability [10]. Age and pre-existing comorbidities weigh heavily in mortality prediction models [37], thus leading to lower discrimination of severity of these models in older patients [38]. We observed a difference between observed mortality and probability of mortality using SAPS-3 which suggests that this score is not suited for older patients.

We found that when adding loss of functional status with mortality as an outcome, admission in an IMCU was no longer associated with this outcome. After propensity score matching, admission to an AMW or an ICU were associated with a higher risk of death or loss of ADL, in comparison with IMCU admission. Although it was not the study’s primary outcome, this finding could encourage to perform specific studies. In all three destinations, age, baseline ADL and severity were similarly associated with this outcome, which could suggest that other unmeasured factors could be involved, such as post-critical care management. Loss in functional ability is a reversible factor that can be prevented during and after hospital stay [39, 40]. A prospective observational study documented that older patients had worse health-related quality of life over time, while younger patients showed spontaneous improvement [14]. This finding suggests that studies evaluating the benefit of early rehabilitation specifically tailored for older population could be of interest. Post-ICU management could, therefore, possibly influence patient outcomes in the older critically ill population.

Large studies evaluating quality of life after a critical condition are scarce. In a study of 23 ICU survivors aged ≥ 80 years, quality of life (using WHOQOL-BREF and WHOQOL-OLD) was similar or better than that of a general population matched on age and gender [15]. On the other hand, among 54 patients aged ≥ 75 years prospectively followed-up after ICU admission, quality of life evaluated using SF-36 was equal or better than that of the general population, except for physical functioning, vitality and mental health SF-36 components [41]. To summarise, large studies evaluating long-term autonomy and quality of life after ICU admission are scarce, and use various scales, thus limiting reproducibility and comparability. Our study is the first to have prospectively used, on a large cohort, both validated and reproducible scales. We found that physical and mental SF-12 was lowest among patients hospitalised in an AMW, although a variation of one point on a 56-point scale is not clinically relevant. Physical and mental SF-12 in our cohort was similar to those of other cohorts of frail, older patients living in the community or in care homes [42–45]. Therefore, after a critical condition, quality of life seems comparable among the 3 hospital destinations, and similar to that of the general older population [42–45].

The present study has several strengths. This study included patients in real-life settings, with high age (median 85 years), multiple comorbidities and high severity at admission. The cohort originates from a prospective multicentre study, with many patients and a low loss of follow-up, thereby limiting bias [25]. Our study used a pragmatic clinical outcome which is 6-month survival and loss of 1-point ADL. Robust, validated and reproducible scales of functional ability and quality of life were used. Our results were confirmed using three different statistical models.

Several limitations should be taken into account. This study was a post-hoc analysis of a prospective study that was not designed for this analysis. Specific studies should be designed to confirm our findings. We did not assess frailty nor decisions of withdrawal of life sustaining therapy, which are two important prognostic factors in the older population [10, 13]. We did not have information on the type of IMCU the patient was hospitalised (general or specialised high dependency units), thus adding heterogeneity in the IMCU group. It cannot be excluded that patients admitted in an ICU or an AMW have more cumulative number of organ failures at admission, than those admitted in an IMCU. Although we have adjusted on SAPS-3 and admission diagnosis, and matched patients according to baseline characteristics using a propensity score, no adjustment is perfect and one cannot exclude a potential indication bias. We also did not assess information on patient movement between each ward, and patients initially hospitalised in an AMW or IMCU could have then been transferred in an ICU, which could be a source of bias. It is possible that data on post-critical care management could influence patient outcome as well. Indeed, our aim was to find predictive factors of poor outcomes at admission, to help the clinician when making triage decisions on which ward to admit a critically ill older patient. A visualization of the post-ICU care trajectory of older patients is a key variable to take into account, as a specific post-ICU geriatric approach, including prevention of geriatric-specific complications and closer follow-up, could ameliorate both long-term survival and functional status.

## Conclusions

From our available data, patients admitted to an AMW had a worse 6-month survival than those hospitalised in an IMCU, with no difference of 6-month survival between IMCU and ICU admission. Mental and physical SF-12 was lowest in patients admitted in an AMW, suggesting that admission in an ICU or IMCU does not alter long-term quality of life. IMCU admission was no longer associated with outcomes when measured using a composite criterion encompassing both loss of ADL and 6-month mortality. Specific studies should be performed to confirm our findings, taking into account the number of organ failures and limitation of life sustaining therapy. In the context of aging of the population with potential tension in critical care capacity, these results could raise the question of the benefit of opening specific geriatric IMCUs.

## Supplementary Information

**Additional file 1: Table S1.** Factors associated with 6-month overall survival according to hospital destination—multivariate analysis. **Table S2.** Multivariate analysis of factors associated with 6-month mortality or loss of one-point ADL at 6 months according to hospital destination. **Table S3.** Multivariate analysis of factors associated with 6-month overall survival using multiple imputation for missing values. **Table S4.** Comparison of 6-month overall survival according to orientation after propensity score matching. **Table S5.** Comparison of mortality or loss of one-point ADL at 6 months according to orientation after propensity score matching.

## Abbreviations

ADL: Activity of daily living
AMW: Acute medical ward
ICU: Intensive care unit
IMCU: Intermediate care unit
LST: Life-sustaining treatment
SAPS-3: Simplified Acute Physiology Score 3
SF-12: 12-Item Short-Form Health Survey
