# Supplementary material for: Impact on 6-month outcomes of hospital trajectory in critically ill older patients: analysis of the ICE-CUB2 clinical trial
Source: Ann Intensive Care. 2022 Jul 11;12:65. doi: 10.1186/s13613-022-01042-4 (PMC9274629; doi:10.1186/s13613-022-01042-4)
Supplement: Supplementary file 1 — Additional file 1: Table S1. Factors associated with 6-month overall survival according to hospital destination—multivariate analysis. Table S2. Multivariate analysis of factors associated with 6-month mortality or loss of one-point ADL at 6 months according to hospital destination. Table S3. Multivariate analysis of factors associated with 6-month overall survival using multiple imputation for missing values. Table S4. Comparison of 6-month overall survival according to orientation after propensity score matching. Table S5. Comparison of mortality or loss of one-point ADL at 6 months according to orientation after propensity score matching. [file 13613_2022_1042_MOESM1_ESM.doc]

**Table S1. Factors associated with 6-month overall survival according to hospital destination – multivariate analysis**

| **Variables** | | **ICU**  **HR (95% CI) *^a^*** | **IMCU**  **HR (95% CI) *^a^*** | **Acute medical ward**  **HR (95% CI) *^a^*** |
| --- | --- | --- | --- | --- |
| **Age (per one-point increase)** | | 1.04 (1.02-1.06)* | 1.11 (1.06-1.16)* | 1.02 (0.99-1.05) |
| **Male sex (vs female)** | | 1.10 (0.95-1.27) | 1.57 (0.99-2.49) | 1.12 (0.81-1.54) |
| **Presence of comorbidities** | | 1.16 (0.91-1.46) | 0.78 (0.55-1.11) | 1.08 (0.73-1.59) |
| **ADL (per one-point decrease)** | | 1.19 (1.05-1.34)* | 1.76 (1.21-2.56)* | 1.06 (0.93-1.20) |
| **SAPS-3 (per one-point increase)** | | 1.04 (1.03-1.05)* | 1.05 (1.03-1.08)* | 1.06 (1.05-1.07)* |
| **Admission diagnosis (ref: cardiac disorder)** | |  |  |  |
| Surgery  Coma  Respiratory failure  Gastrointestinal tract disorder  Shock  Multiple trauma with no surgery  Acute kidney injury  Other | | 0.98 (0.55-1.75)  0.87 (0.46-1.66)  0.77 (0.47-1.26)  0.64 (0.33-1.24)  0.74 (0.43-1.26)  1.64 (0.66-4.04)  0.85 (0.49-1.48)  0.77 (0.45-1.31) | 1.66 (0.46-5.98)  1.53 (0.79-2.96)  1.14 (0.44-2.98)  0.83 (0.50-1.40)  1.32 (0.54-3.22)  0.77 (0.42-1.43) | 1.43 (0.64-3.18)  1.84 (1.16-2.93)*  1.12 (0.67-1.89)  0.27 (0.08-0.89)*  0.88 (0.51-1.51)  4.41 (0.39-49.75)  1.36 (0.58-3.22)  1.03 (0.59-1.82) |
| **Time of emergency department visit (day vs night)** | | 0.97 (0.7-1.34) | 1.5 (0.84-2.69) | 0.72 (0.46-1.13) |
| **Emergency physician experience (ref: resident)** |  | | | |
| **<2 years**  **≥2 years** | | 1.48 (1.00-2.19)  1.43 (1.08-1.89)* | 1.33 (0.52-3.39)  1.12 (0.71-1.78) | 0.88 (0.58-1.33)  1.12 (0.66-1.89) |
| **ICU physician experience at admission (ref: resident)** |  | | | |
| **<2 years**  **≥2 years** | | 1.54 (1.05-2.27)*  1.4 (1.00-1.95)* | 0.9 (0.58-1.40)  0.47 (0.28-0.79)* | 0.58 (0.25-1.3)  0.64 (0.34-1.18) |

*^a^ HR gives the increase of the risk of death per each unit increase for continuous variables and for one specific category versus a reference category for categorical variables (HR>1: the variable is associated with and increased risk of death or decreased survival).*

*95% CI: 95% confidence interval, ADL: Activity of daily living, ICU: intensive care unit, IMCU: intermediate care unit, HR: hazard ratio, ref: reference, SAPS-3: Simplified Acute Physiology Score 3.*

** p-value <0.05*

**Table S2. Multivariate analysis of factors associated with 6-month mortality or loss of one-point ADL at 6 months according to hospital destination.**

| **Variables** | | **ICU**  **HR (95% CI) *^a^*** | **IMCU**  **HR (95% CI) *^a^*** | **Acute medical ward**  **HR (95% CI) *^a^*** |
| --- | --- | --- | --- | --- |
| **Age (per one-point increase)** | | 1.01 (1.01-1.02)* | 1.02 (1.01-1.03)* | 1.01 (1.01-1.02)* |
| **Male sex (vs female)** | | 0.98 (0.93-1.04) | 0.84 (0.74-0.95)* | 1.02 (0.93-1.11) |
| **Presence of comorbidities** | | 1.04 (0.98-1.11) | 1.05 (0.92-1.21) | 1.06 (0.97-1.16) |
| **ADL (per one-point decrease)** | | 1.04 (0.99-1.09) | 1.09 (0.99-1.21) | 1.00 (0.95-1.04) |
| **SAPS-3 (per one-point increase)** | | 1.01 (1.01-1.01)* | 1.01 (1.00-1.02)* | 1.01 (1.01-1.02)* |
| **Initial diagnosis (ref: cardiac disorder)** |  | | | |
| Surgery  Coma  Respiratory failure  Gastrointestinal tract disorder  Shock  Multiple trauma with no surgery  Acute kidney injury  Other | | 0.89 (0.72-1.11)  0.96 (0.83-1.10)  0.93 (0.84-1.04)  1.01 (0.85-1.20)  0.91 (0.81-1.02)  0.96 (0.67-1.37)  0.89 (0.77-1.03)  0.89 (0.77-1.04) | 0.95 (0.66-1.37)  1.00 (0.84-1.18)  1.01 (0.75-1.36)  0.73 (0.57-0.94)*  0.92 (0.69-1.22)  0.89 (0.76-1.04) | 1.16 (0.80-1.67)  1.26 (1.08-1.48)*  1.16 (1.00-1.34)*  0.87 (0.69-1.09)  0.97 (0.83-1.14)  1.10 (0.60-2.01)  1.09 (0.84-1.41)  0.97 (0.81-1.16) |
| **Time of emergency department visit (day vs night)** | | 0.97 (0.89-1.06) | 1.11 (0.94-1.32) | 0.96 (0.86-1.07) |
| **Emergency physician experience (ref: resident)** |  | | | |
| **<2 years**  **≥2 years** | | 1.07 (0.96-1.19)  1.00 (0.91-1.09) | 1.07 (0.91-1.27)  1.06 (0.9-1.25) | 1.02 (0.88-1.20)  1.03 (0.89-1.18) |
| **ICU physician experience at admission (ref: resident)** |  | | | |
| **<2 years**  **≥2 years** | | 1.00 (0.86-1.17)  0.99 (0.87-1.14) | 0.94 (0.73-1.20)  0.96 (0.78-1.18) | 0.91 (0.74-1.12)  0.98 (0.82-1.19) |

*^a^ HR gives the increase of the risk of death per each unit increase for continuous variables and for one specific category versus a reference category for categorical variables (HR>1: the variable is associated with and increased risk of death or decreased survival)*

*95% CI: 95% confidence interval, ADL: Activity of daily living, ICU: intensive care unit, IMCU: intermediate care unit, HR: hazard ratio, ref: reference, SAPS-3: Simplified Acute Physiology Score 3.*

** p-value <0.05*

**Table S**3. Multivariate analysis of factors associated with 6-month overall survival using multiple imputation for missing values.

| **Variables** | **Hazard Ratio**  **(95% CI)a** | **p-value** |
| --- | --- | --- |
| Hospital destination (vs IMCU)  AMW  ICU | 1.35 (1.11-1.63)  1.19 (0.99-1.44) | 0.002  0.069 |
| Age (per one-point increase) | 1.04 (1.03-1.05) | <0.001 |
| Male sex (vs female) | 1.13 (1.01-1.27) | 0.037 |
| Presence of comorbidities | 1.07 (0.94-1.21) | 0.313 |
| SAPS-3 (per one-point increase) | 1.05 (1.04-1.05) | <0.001 |
| ADL (per one-point decrease) | 1.16 (1.08-1.26) | <0.001 |
| Admission diagnosis (ref: cardiac disorder)  Surgery  Coma  Respiratory failure  Gastrointestinal tract disorder  Shock  Multiple trauma with no surgery  Acute kidney injury  Other | 0.93 (0.6-1.44)  1.41 (1.13-1.77)  0.91 (0.75-1.1)  0.75 (0.56-1.02)  0.79 (0.63-0.97)  1.13 (0.53-2.42)  0.92 (0.68-1.24)  0.82 (0.64-1.04) | 0.757  0.002  0.325  0.07  0.028  0.757  0.58  0.101 |

*Multiple imputation used predictive mean matching for continuous variables, logistic regression for binary data, and polytomous regression for (unordered) categorical data. The cumulative baseline hazard was approximated by the Nelson-Aalen estimator and included in the imputation model. Fifty imputations were drawn. aCox models were estimated in each imputed datasets and estimates were combined using Rubin's rules to give an overall estimate of parameters and corresponding variance‐covariance matrix.*

**Table S4. Comparison of 6-month overall survival according to orientation after propensity score matching**

|  | **Six-month overall survival** | |
| --- | --- | --- |
| **Variable** | **HR (95%CI)a** | **p-value** |
| AMW (vs IMCU)b | 1.76 (1.23-2.53) | 0.002 |
| ICU (vs IMCU)c | 1.5 (1.07-2.10) | 0.02 |

*Propensity score was computed using the following variables:* *age, gender, invalidating illness, ADL, SAPS 3, admission diagnosis, living situation, experience of the ED physician, experience of the intensive care physician; then patients were matched on the propensity score with a ratio 1:1. a Using Cox model, results show the Hazard ratio of risk of death at 6 months. Analysis used the twang package from R. b 233 patients admitted to AMW matched to 233 IMCU patients with complete data. c 233 patients admitted to ICU matched to 233 IMCU patients with complete data. MatchIt package was used, using the nearest neighbour matching.*

*AMW: acute medical ward; ICU: intensive care unit; IMCU: intermediate care unit; HR: hazards ratio; OR: odds ratio*

**Table S5. Comparison of mortality or loss of one-point ADL at 6 months according to orientation after propensity score matching**

|  | **Death or loss of one-point ADL at six months** | |
| --- | --- | --- |
| **Variable** | **OR (95%CI)a** | **p-value** |
| AMW (vs IMCU)b | 1.12 (1.03-1.22) | 0.011 |
| ICU (vs IMCU)c | 1.15 (1.06-1.26) | 0.001 |

*Propensity score was computed using the following variables: age, gender, invalidating illness, ADL, SAPS 3, admission diagnosis, living situation, experience of the ED physician, experience of the intensive care physician; then patients were matched on the propensity score with a ratio 1:1. a* *Using logistic regression model, results show the Odds ratio of death or loss of one-point ADL at six months. b 233 patients admitted to AMW matched to 233 IMCU patients with complete data. c 233 patients admitted to ICU matched to 233 IMCU patients with complete data. MatchIt package was used, using the nearest neighbour matching.*

*AMW: acute medical ward; ICU: intensive care unit; IMCU: intermediate care unit; HR: hazards ratio; OR: odds ratio.*
